# OP42 Cost-Utility Analysis Of Axicabtagene Ciloleucel (Yescarta) Versus Standard Care For Relapsed Or Refractory Large B-Cell Lymphoma – Health Payer Perspective

**DOI:** 10.1017/S0266462325101001

**Published:** 2025-12-29

**Authors:** Danielle Stringer, Ming Min, Konstance Nicolopoulos, Magdalena Moshi, Thomas Vreugdenburg

## Abstract

**Introduction:**

Axicabtagene ciloleucel (axi-cel) is a chimeric antigen receptor T-cell (CAR-T) therapy approved for relapsed or refractory (r/r) diffuse large B-cell lymphoma (DLBCL) and r/r primary mediastinal B-cell lymphoma (PMBCL). CAR-T therapy is a type of personalized medicine that offers potentially curative options for patients with limited treatment alternatives. This study evaluated the cost effectiveness of axi-cel as a third-line therapy compared with standard care (SoC) in Switzerland.

**Methods:**

A cost-utility analysis was conducted using a healthcare payer perspective. Only direct medical costs in 2023 CHF were considered. Health outcomes were measured in life years and quality-adjusted life years. Both costs and outcomes were discounted at three percent per annum. A lifetime horizon was used to capture differences in costs and outcomes. A hybrid decision tree and three-state partitioned survival model simulated outcomes using survival data from single-arm studies and extrapolated Kaplan-Meier curves. Comparators included SoC options such as salvage chemotherapy. Sensitivity analyses explored uncertainty related to survival assumptions, time horizons, and product costs. The research project was funded by the Swiss Federal Office of Public Health.

**Results:**

Axi-cel demonstrated significant long-term survival benefits for DLBCL and PMBCL, compared with SoC, with improved overall survival and progression-free survival. However, the high costs of CAR-T therapies, particularly the product itself, posed substantial economic challenges. Adverse events such as cytokine release syndrome and immune effector cell-associated neurotoxicity syndrome were prevalent and contributed to treatment costs. Sensitivity analyses confirmed the cost effectiveness was highly dependent on survival extrapolations and pricing assumptions.

**Conclusions:**

Axi-cel provided substantial survival benefits in DLBCL and PMBCL; however, its high economic burden necessitates careful cost management and monitoring. Due to limited comparative evidence, naïve comparisons were relied upon to estimate the incremental benefit of axi-cel, introducing high levels of uncertainty into the results. Further research should refine long-term survival projections and optimize pricing models to enhance affordability. These findings guide healthcare policy on axi-cel reimbursement and adoption for r/r DLBCL and PMBCL.

